# Carbon nanotubes: are they dispersed or dissolved in liquids?

**DOI:** 10.1186/1556-276X-6-136

**Published:** 2011-02-11

**Authors:** Kurt Ernst Geckeler, Thathan Premkumar

**Affiliations:** 1 Department of Nanobio Materials and Electronics, World-Class University (WCU), 1 Oryong-dong, Buk-gu, Gwangju 500-712, South Korea; 2 Department of Materials Science and Engineering, Gwangju Institute of Science and Technology (GIST), 1 Oryong-dong, Buk-gu, Gwangju 500-712, South Korea

## Abstract

Carbon nanotubes (CNTs) constitute a novel class of nanomaterials with remarkable applications in diverse domains. However, the main intrincsic problem of CNTs is their insolubility or very poor solubility in most of the common solvents. The basic key question here is: are carbon nanotubes dissolved or dispersed in liquids, specifically in water? When analyzing the scientific research articles published in various leading journals, we found that many researchers confused between "dispersion" and "solubilization" and use the terms interchangeably, particularly when stating the interaction of CNTs with liquids. In this article, we address this fundamental issue to give basic insight specifically to the researchers who are working with CNTs as well asgenerally to scientists who deal with nano-related research domains.

## 

Among the various nanomaterials, CNTs gained widespread attention owing to their exceptional properties, good chemical stability, and large surface area [1,2]. CNTs are extremely thin tubes and feature an extremely enviable combination of mechanical, thermal, electrical, and optical properties. Their size, shape, and properties construct them as prime contenders for exploiting the growth of a potentially revolutionary material for diverse applications.

Nevertheless, the main intrinsic drawback of CNTs is their insolubility or extremely poor solubility in most of the common solvents due to their hydrophobicity, thus creating it tricky to explore and understand the chemistry of such material at the molecular level and device applications. Though diverse approaches [3] have been introduced to improve the dispersion of CNTs in different solvents including water, challenges still remain in developing simple, green, facile, and effective strategies for a large-scale production of CNT dispersions. To this end, in many studies a wide range of agents have been used. To give a few examples: solvents [4], biopolymers [5], and surfactants [6]. Meanwhile, when analyzing the scientific research articles published in various leading journals, regarding the dispersion of CNTs, it is really puzzling owing to the usage of different terminologies with respect to the dispersion of CNTs. Most of the studies indicated "dispersion"; however, considerable quantities of articles were published with the term "solubilization", which can be evidently seen from the literature analysis [7]. Hence, many researchers confound "dispersion" and "solubilization" and use the terms interchangeably, especially when describing the interaction of CNTs with solvents. Many scientists have mentioned that CNTs can be "solubilized in water or organic solvents" by means of polymers and/or surfactants, which is ambiguous. It is evident that there is, as a result of that, a lot of confusion regarding this fundamental matter. The basic and fundamental key question here is: are CNTs dissolved or dispersed in a liquid?

Basically, "dispersion" and "solubilization" are different phenomena. Dispersion and solubilization can be defined as "*a system, in which particles of any nature *(*e.g., solid, liquid, or gas*) *are dispersed in a continuous phase of a different composition *(*or state*)" [8] and a "*process, by which an agent increases the solubility or the rate of dissolution of a solid or liquid solute" *[9], respectively. Hence, in general, the dispersion of solute *particles *in solvents leads to the formation of colloids or suspensions, and solutions may be obtained as a result of solubilization of solute *molecules or ions *in the specified solvent. Furthermore, dispersion is mostly related to solute *particles*, whereas solubility or solubilization is generally connected with solute *molecules or ions*.

The main differences between a colloid and a solution are: A solution is homogenous and remains stable and does not separate after standing for any period of time. Further it cannot be separated by standard separation techniques such as filtration or centrifugation. A solution looks transparent and it can transmit the light. Also, solutions contain the solute in a size at the molecular or ionic level, typically less than 1 nm or maximum a few nm in all dimensions. A colloid is a mixture with particles sizes between 1 and 1000 nm in at least one dimension. It is opaque, non-transparent, and the particles are large enough to scatter light. Colloids are not as stable as solutions and the dispersed particles (comparatively larger-sized particles) may be conveniently separated by standard separation techniques such as (ultra)centrifugation or filtration. Frequently, dispersed particles in colloidal systems may slowly agglomerate owing to inter-particle attractions over prolonged periods of time and, as a result, colloidal dispersions may form flocs or flakes.

As far as CNTs are concerned, even though the diameter of the tubes is in the nanometer range (approximately between 0.4 and 3 nm for single-walled carbon nanotubes, and 1.4 and 100 nm for multi-walled carbon nanotubes) [10], their length can be up to several micrometers to millimeters. Further, it is a well-known fact that CNTs are not equal in size with respect to both diameter and length. Hence, the result of dispersion techniques mostly used and adopted to produce well-dispersed CNTs in either aqueous and/or organic media are typically dispersions of differently sized tubes. Consequently, based on the definition [6,7] and the abovementioned points, the mixture of CNTs and water or organic solvents, whether in the presence or non-presence of dispersing agents such as surfactants or polymers, is just a colloidal dispersion and not a solution. Figure 1 shows the schematic illustration for the formation of dispersed CNTs in a liquid with the aid of a dispersing agent. Simultaneously, the dispersion can result in a debundling or individualization of the bundled CNTs.

**Figure 1:**
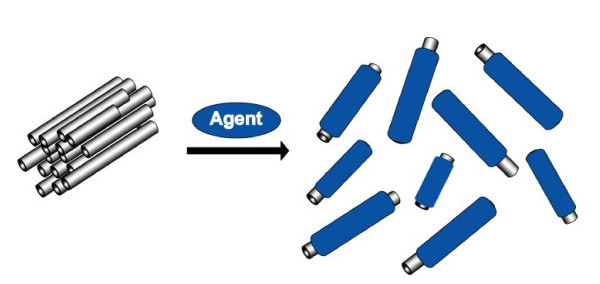
**Schematic showing the transition of the bundled to the individualized, dispersed state of carbon nanotubes in a liquid with the aid of a dispersing agent**.

Therefore, "solubilization" is a process to achieve a stable solution, whereas "dispersion" is a form of colloidal system. Here we conclude that the mixture obtained by using CNTs and a liquid medium (water or organic solvents) with or without surfactants or polymers is a dispersion of CNTs in the medium, but not a solution. Further, in our opinion, one cannot solubilize CNTs in water or organic solvents. Hence, we recommend to restrict the use of the term "solubilization" or "solution," instead we should use the term "dispersion" or "colloid," when dealing with CNTs. Further, we think that this should be also applicable for nanoparticles of comparable dimensions such as metal and metal oxide nanoparticles, polymer nanoparticles, etc., if the criteria of the definitions given above are fulfilled.

In short, the term "dispersion" should exclusively be used as far as CNTs are concerned, and the use of the term "solution" should be avoided or restricted.

## Abbreviations

CNT: carbon nanotubes.

## Competing interests

The authors declare that they have no competing interests.

## Authors' contributions

KEG designed the the article as well as corrected the manuscript with critical comments. TP drafted the article and did the literature survey and analysis. Both authors read and approved the final manuscript.
